# Magnetic resonance imaging for the assessment of cardiac compression caused by a giant hiatal hernia

**DOI:** 10.1093/ehjcr/ytae070

**Published:** 2024-02-24

**Authors:** Satoshi Kurisu, Hitoshi Fujiwara

**Affiliations:** Department of Cardiology, Hiroshimanishi Medical Center, Otake, Japan; Department of Cardiology, Hiroshimanishi Medical Center, Otake, Japan

An 86-year-old woman with an untreated giant hiatal hernia presented with a 3-month history of exertional dyspnoea.^1^ A chest radiograph showed multiple abnormal gas shadows overlapping the heart (*Figure 1*, Panel *A*). Right-sided pleural effusion suggested heart failure. Computed tomography revealed a giant hiatal hernia with most of the stomach escaping into the thoracic cavity (*Figure 1*, Panel *B*). The hernia was located immediately adjacent to the left ventricle (LV) and atrium. Cine magnetic resonance imaging (MRI) was performed to assess the effects of the giant hiatal hernia on LV function during a cardiac cycle (*Figure 1*, Panel *C*). Serial LV short-axis images at the following four time points are shown with the schematic diagram of the LV pressure-volume curve. First, the LV free wall was compressed from the hiatal hernia at the end of the LV filling with a characteristically inverted D-shaped LV cavity (lower right corner, arrow). Next, LV pressure increased at the end of isovolumic contraction, thereby producing a circular LV cavity (upper right corner). Subsequently, LV volume decreased with a circular and smaller LV cavity at the end of LV ejection (upper left corner). Finally, LV pressure decreased at the end of isovolumic relaxation, thereby reproducing an inverted D-shaped LV cavity (lower left corner, arrow). Despite the significant cardiac compression visualized on MRI, N-terminal pro-brain natriuretic peptide level (41 pg/mL) was not elevated, and this may be due to impaired LV stretching.^2^ It was suggested that dyspnoea was due to mechanical effects of the giant hiatal hernia, which caused impaired LV filling as well as reduced lung capacity. She refused surgical treatment and was followed up with home oxygen therapy.^3^

**Figure 1:**
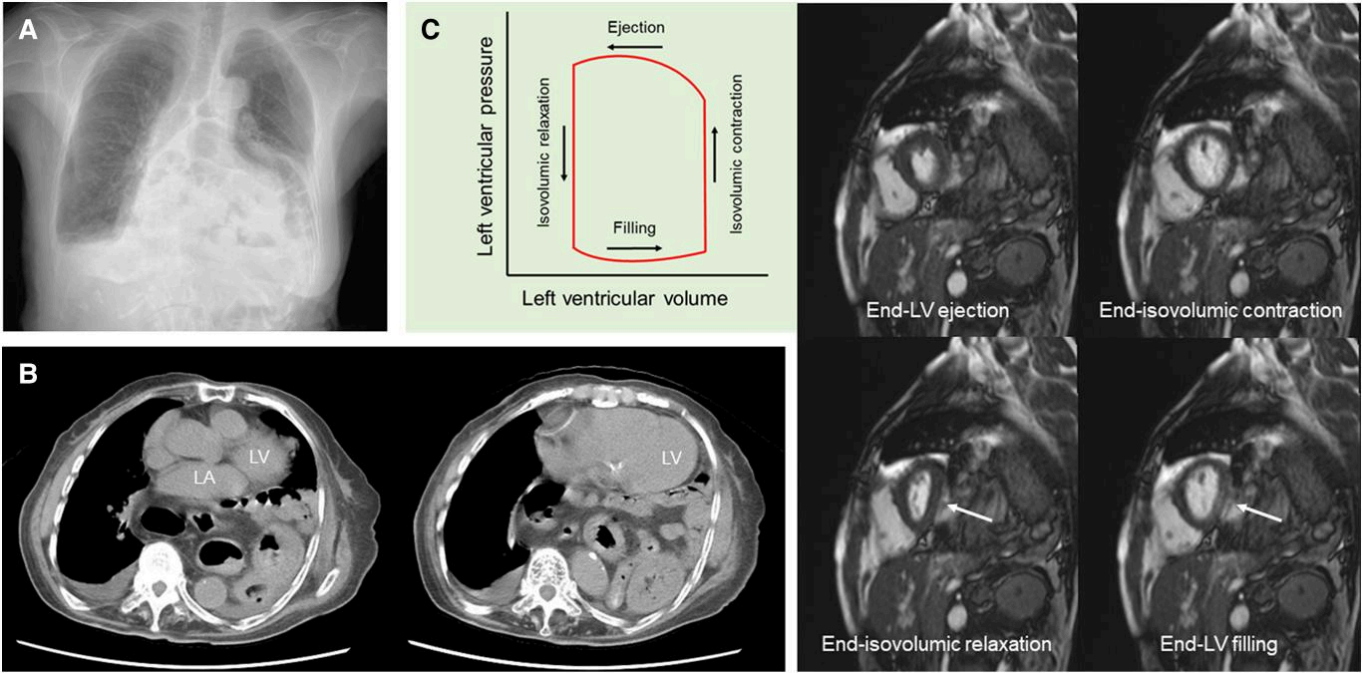
A chest radiograph showing multiple abnormal gas shadows overlapping the heart and right-sided pleural effusion (Panel *A*). The hernia located immediately adjacent to the left ventricle and atrium (Panel *B*). Serial left ventricle short-axis images at the four time points with the schematic diagram of left ventricle pressure-volume curve (Panel *C*).

Most patients with hiatal hernia are asymptomatic. Typical symptoms include gastroesophageal disease, whereas heart failure as a main symptom is rare. Clinicians should recognize giant hiatal hernia as a possible cause of heart failure.

## Data Availability

The data that support the findings of this study are available from the corresponding author upon reasonable request.
